# Rapid progression of monoclonal gammopathy of undetermined significance to cardiac amyloidosis with intracardiac thrombus: Unveiling via cardiac magnetic resonance

**DOI:** 10.1016/j.radcr.2025.02.022

**Published:** 2025-03-08

**Authors:** Hadeel A. Al Kayed, Younes Hamam, Noor Al Kayed, Ali O. Alajarmeh

**Affiliations:** a Faculty of Medicine, The University of Jordan, Amman, Jordan; b Deparment of Cardiac Radiology, Royal Jordanian Medical Services, Amman, Jordan

**Keywords:** Cardiac amyloidosis, Monoclonal gammopathy of undetermined significance, Supraventricular tachycardia, Transthoracic echocardiography, Cardiac magnetic resonance, Intracardiac thrombus, Restrictive cardiomyopathy

## Abstract

We present the case of a 67-year-old male diagnosed with Monoclonal Gammopathy of Undetermined Significance (MGUS) who developed progressive peripheral edema and abdominal distension, raising concerns for right-sided heart failure. Initial investigations, including electrocardiography and transthoracic echocardiography (TTE), revealed findings suggestive of restrictive diastolic dysfunction, including low-voltage QRS complexes, concentric left ventricular hypertrophy, left atrial enlargement, and valvular thickening, despite a preserved ejection fraction (EF) of 62%. These findings raised suspicion for AL-type cardiac amyloidosis in the context of the patient's recent MGUS diagnosis. Although no thrombus was detected on TTE, cardiac magnetic resonance imaging (CMR) conducted 2 weeks later demonstrated findings supportive of cardiac amyloidosis, such as myocardial infiltration and increased extracellular volume, and identified a left atrial appendage thrombus which was not visualized on the initial TTE. This case highlights the diagnostic superiority of CMR over TTE in detecting intracardiac thrombi. While transesophageal echocardiography (TEE) remains the gold standard for thrombus detection, its semi-invasive nature limits its routine use in initial evaluations. The findings underscore the importance of early utilization of CMR in suspected cardiac amyloidosis cases, emphasizing its comprehensive noninvasive assessment capabilities for guiding timely diagnosis and management.

## Introduction

Monoclonal Gammopathy of Undetermined Significance (MGUS) is a plasma cell disorder marked by the presence of a monoclonal protein in the serum below 3 g/dL, with fewer than 10% plasma cells in the bone marrow, and without clinical features of advanced plasma cell disorders like hypercalcemia, renal failure, anemia, or lytic bone lesions. Although generally benign and asymptomatic, MGUS poses a risk of progression to more severe conditions, such as multiple myeloma or AL amyloidosis, at an estimated annual rate of 1% [1].

The transition from MGUS to AL amyloidosis involves the misfolding of monoclonal light chains, which deposit as amyloid fibrils in various organs, most commonly the kidneys, liver, heart, and peripheral nerves. Cardiac involvement in amyloidosis occurs when amyloid fibrils accumulate within the myocardium, directly causing restrictive cardiomyopathy and diastolic dysfunction, which may progress to heart failure.

The progression from MGUS to cardiac amyloidosis occurring in a short period, as observed in this case, is uncommon and clinically important, as it contrasts with the generally indolent nature of MGUS [2]. In this case, within a few months of MGUS diagnosis, the patient presented with cardiac amyloidosis and the rare finding of an intracardiac thrombus in the absence of atrial fibrillation (Afib). The detection of an intracardiac thrombus in cardiac amyloidosis highlights an elevated risk of thromboembolic events, which may arise from structural and functional abnormalities of the myocardium due to amyloid infiltration [3]. These risks can persist despite normal sinus rhythm and appropriate anticoagulant management, possibly due to altered blood flow dynamics and vascular fragility linked to amyloid deposition. This raises the importance of heightened vigilance and the use of advanced imaging modalities for early detection.

While transthoracic echocardiography (TTE) is the standard tool for evaluating cardiac function, it may not fully capture the extent of myocardial involvement in infiltrative diseases like amyloidosis. In this case, cardiac magnetic resonance (CMR) played a crucial role in providing a detailed myocardial assessment, including fibrosis, edema, and extracellular volume expansion, while also identifying an intracardiac thrombus missed on initial TTE. This emphasizes the importance of advanced imaging for early detection and management, particularly in MGUS patients at risk of AL amyloidosis progression, where timely anticoagulation can be critical in preventing thromboembolic complications.

## Case presentation

A 67-year-old male presented to the cardiology department 3 months after being diagnosed with MGUS. His initial diagnosis had been based on laboratory tests and a bone marrow biopsy conducted due to nonspecific symptoms, including fatigue, a 5 kg weight loss, and shortness of breath. At the time of diagnosis, a cardiac catheterization was performed to evaluate his shortness of breath, but it revealed no abnormalities. No further cardiac assessments were undertaken, as cardiac involvement in MGUS is rare and typically occurs later in the disease course. Upon presentation to our department, he exhibited significant peripheral edema and ascites, prompting further evaluation to investigate potential cardiac involvement.

Electrocardiography (ECG) revealed supraventricular tachycardia (SVT) and low-voltage QRS complexes in leads V1-V4. Laboratory tests revealed significantly elevated levels of proBNP (13,344.2 pg/mL; normal <125 pg/mL) and high-sensitivity troponin (57.0 pg/mL; normal <14 ng/L). Mild anemia was noted with a hemoglobin level of 13 g/dL (normal range: 13.8-17.2 g/dL), along with mild renal impairment reflected by a creatinine level of 1.75 mg/dL (normal range: 0.7-1.3 mg/dL).

Transthoracic echocardiography (TTE) demonstrated concentric left ventricular hypertrophy, a preserved ejection fraction (EF) of 62%, and thickened mitral, aortic, and tricuspid valve leaflets. Additionally, the patient exhibited grade III diastolic dysfunction and mild to moderate mitral valve regurgitation, findings consistent with cardiac amyloidosis.

A CMR performed 2 weeks later revealed decreased diastolic and systolic function of the left ventricle, with an EF of 46.55%, accompanied by concentric hypertrophy most pronounced in the septal wall (thickness ranging from 14.1 mm to 15.6 mm, measured on 4- and 2-chamber views during the end-diastolic phase). The end-diastolic volume (EDV) was measured at 104.14 mL (normal range: 56-78 mL), and the end-systolic volume (ESV) was 55.46 mL (normal range: 19-27 mL), suggestive of compromised ventricular filling and reduced systolic performance.

**Fig. 1:**
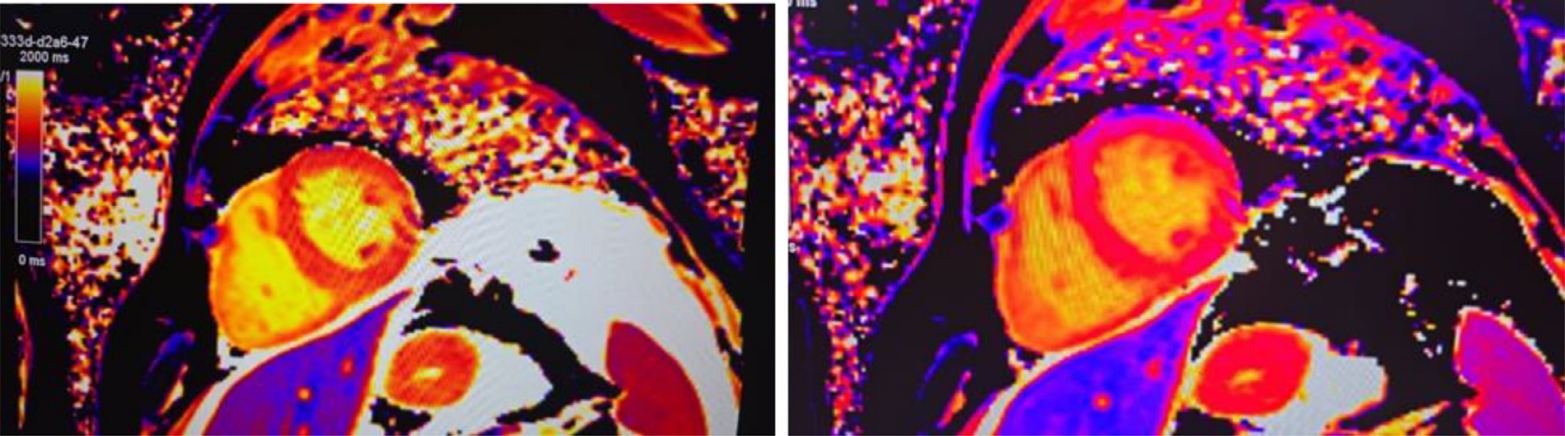
Native T1 map (left) and postcontrast T1 map (right) from cardiac MRI. The native T1 map demonstrates significantly elevated T1 relaxation times (depicted in red and orange) throughout the left ventricular myocardium, indicating extensive amyloid infiltration. The postcontrast T1 map shows elevated postcontrast T1 relaxation times, reflecting extracellular matrix expansion caused by amyloid infiltration.

Abnormal high signal intensities were observed on T1 and T2 myomap images, particularly in the inferior lateral wall. Native T1 myomap (Fig. 1, left) reveals significantly elevated T1 relaxation times throughout the myocardium, consistent with amyloid infiltration; and postcontrast T1 myomap (Fig. 1, right) demonstrates globally elevated relaxation times, reflecting extracellular matrix expansion caused by amyloid deposits. The extracellular volume (ECV) fraction was significantly elevated at 52.3%, reflecting the amyloid burden.

**Fig. 2:**
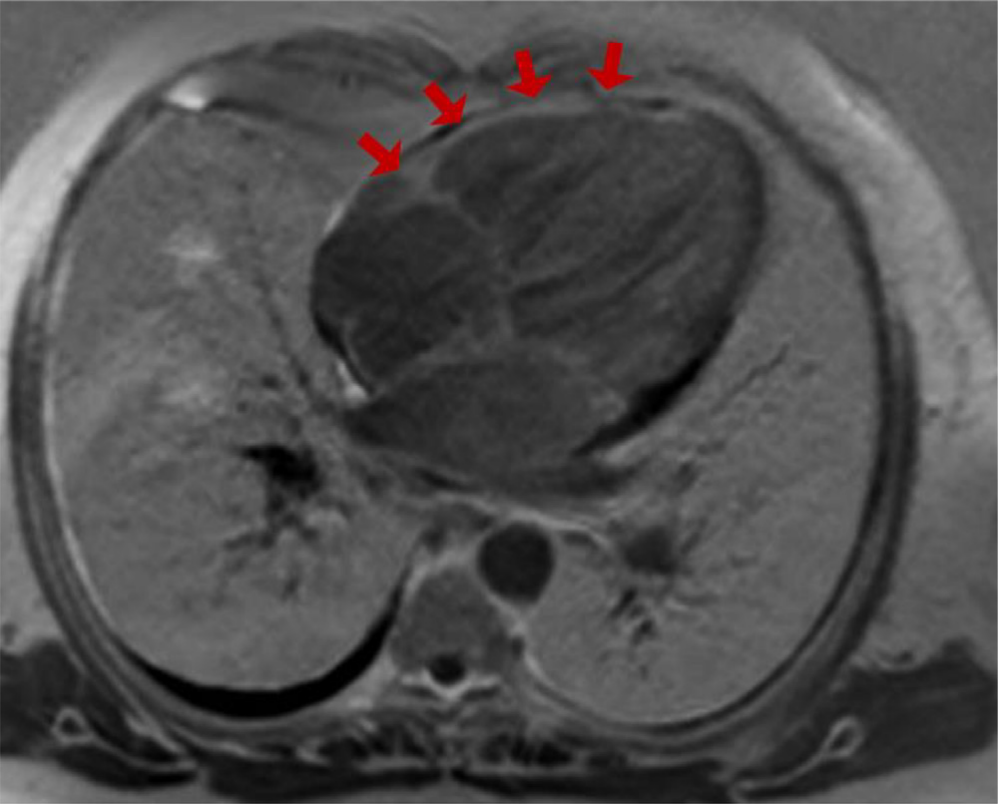
Phase-sensitive inversion recovery (PSIR) image obtained during delayed postcontrast imaging demonstrates patchy mid-wall enhancement in the lateral wall, consistent with amyloid infiltration.

Delayed postcontrast imaging demonstrated patchy mid-wall enhancement in the lateral wall, consistent with amyloid infiltration (Fig. 2). An abnormal nulling pattern of the myocardium, characterized by prolonged nulling time, further confirmed the diagnosis of cardiac amyloidosis.

**Fig. 3:**
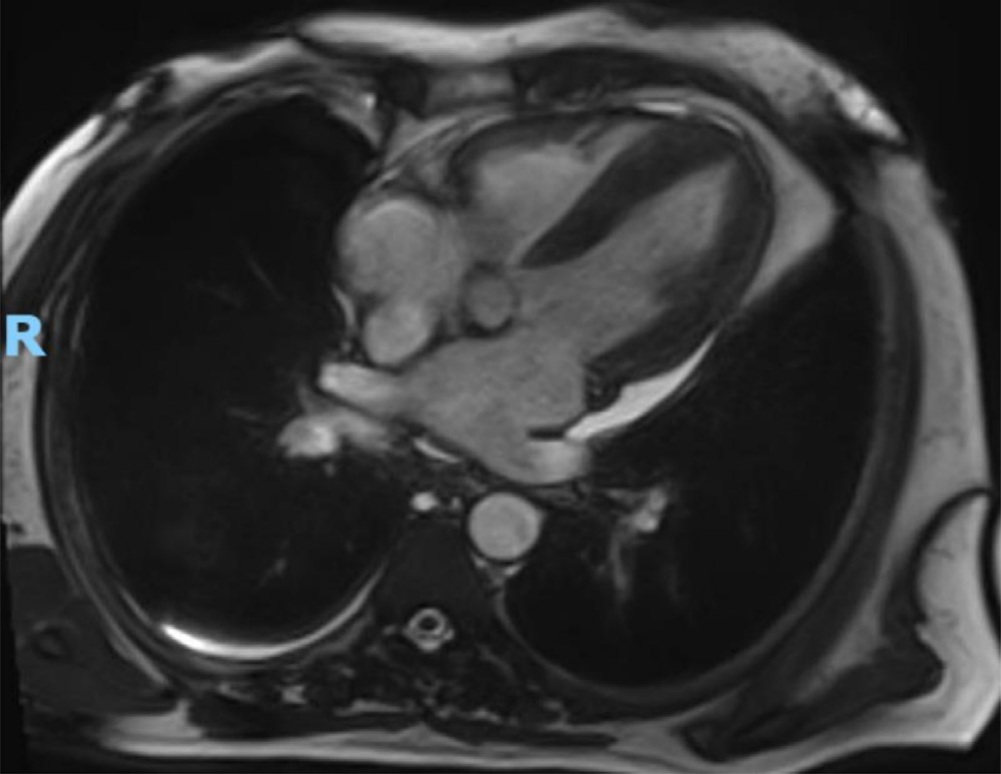
Cine SSFP 4-chamber view demonstrates moderate biatrial enlargement, particularly of the left atrium. The image also shows concentric left ventricular hypertrophy, with mild thickening of the septum and other walls.

**Fig. 4:**
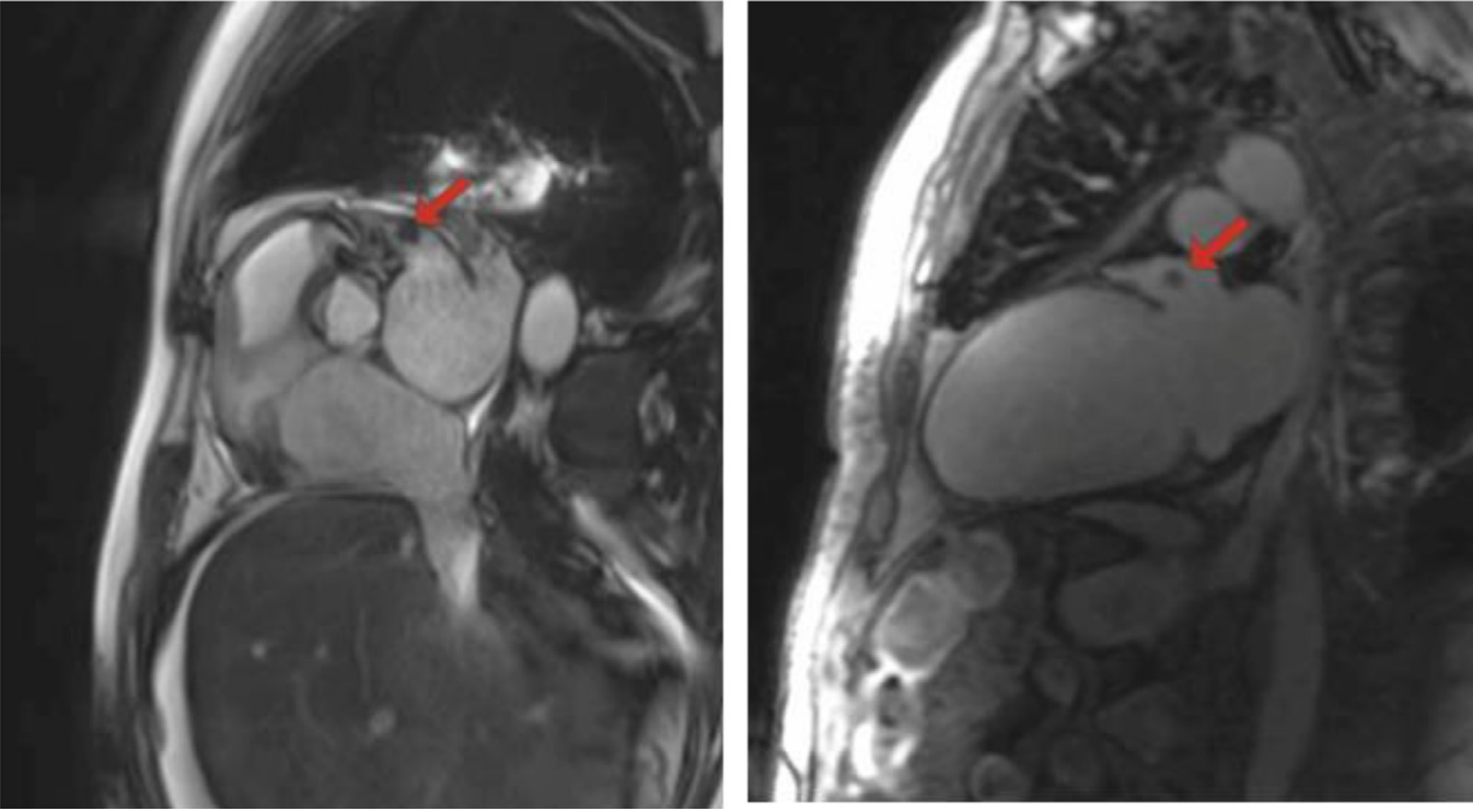
Retrospectively gated cine True FISP sequence focused on the aortic valve (left) demonstrating a left atrial thrombus (red arrow) and T1-native 2-chamber view (right) showing a left atrial thrombus (red arrow).

**Fig. 5:**
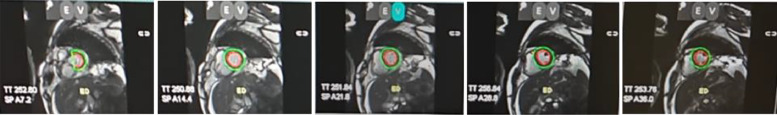
Sequential cine frames from CMR illustrating left ventricular segmentation for functional analysis. The green outline delineates the LV myocardium, while red and yellow contours highlight the chamber volumes during end-diastolic and end-systolic phases. The images reveal concentric LV hypertrophy and a thrombus in the left atrial appendage is visualized as a signal-void region.

Cine Steady-State Free Precession (SSFP) sequence demonstrated moderate biatrial enlargement, particularly of the left atrium, and concentric left ventricular hypertrophy, with mild thickening of the septum and other walls (Fig. 3). Additionally, a left atrial appendage thrombus measuring 9.6 x 8.8 mm was identified as a signal-void area without enhancement on postcontrast images and was also visualized in sequential cine frames as a distinct, noncontractile region (Fig. 4, Fig. 5).

Following stabilization with intravenous diuretics, oxygen therapy, and adjustments to preload and afterload, a long-term management plan was initiated. The regimen included sacubitril/valsartan for heart failure management, bisoprolol and amiodarone for SVT control and rhythm management, and diuretics (furosemide and spironolactone) for fluid overload. Rosuvastatin was added for cardiovascular protection. The decision to initiate amiodarone was based on clinical concern regarding the elevated risk of arrhythmias in the setting of amyloidosis, despite no evidence of atrial fibrillation at the time. This preventive approach was deemed necessary given the high thromboembolic risk associated with cardiac amyloidosis.

In considering the differential diagnosis, other causes of restrictive cardiomyopathy, such as hypertensive heart disease or primary valvular disorders, were evaluated but ruled out based on the distinct pattern of restrictive dysfunction, low-voltage QRS complexes, and confirmed MGUS. This case highlights the critical role of multimodal imaging, particularly CMR, in the comprehensive assessment and management of cardiac amyloidosis.

## Discussion

### MGUS and rapid progression to cardiac amyloidosis

MGUS is defined by the presence of monoclonal protein in the serum at concentrations below 3 g/dL, fewer than 10% plasma cells in the bone marrow, and no associated complications such as hypercalcemia, renal dysfunction, anemia, bone lesions, or amyloid deposits. Although progression from MGUS to conditions like multiple myeloma or AL amyloidosis is generally slow [1], this case highlights an atypically rapid transition to cardiac amyloidosis within just 3 months of diagnosis, emphasizing the importance of close clinical surveillance.

### Ejection fraction discrepancy: echocardiography vs CMR

Cardiac amyloidosis is characterized by restrictive cardiomyopathy and diastolic dysfunction, which can result in heart failure with preserved systolic function (EF> 50%). This case presents 2 different EF, with echocardiography showing a preserved EF of 62% and CMR revealing a reduced EF of 46%. This discrepancy arises from differences in how the 2 modalities assess myocardial function. Echocardiography may overestimate EF in cases where regional wall motion abnormalities or myocardial infiltration are present but not fully captured. In contrast, CMR provides a more precise and comprehensive assessment, especially in infiltrative diseases like amyloidosis. The lower EF seen on CMR reflects the true extent of amyloid infiltration, indicating compromised systolic function. Clinically, this discrepancy emphasizes the need for advanced imaging to accurately assess cardiac dysfunction and guide management, as CMR findings prompted more aggressive heart failure treatment in this patient.

### CMR findings and the role in diagnosing amyloidosis

CMR provides detailed myocardial tissue characterization, making it a cornerstone in the diagnosis of cardiac amyloidosis. Diffuse subendocardial or transmural late gadolinium enhancement (LGE) is a hallmark finding in cardiac amyloidosis, representing amyloid infiltration within the myocardium [4]. Additionally, T1 mapping enables quantitative assessment of myocardial changes, with elevated native T1 values correlating with amyloid burden and severity of disease [4,5]. Notably, noncontrast T1 mapping has shown superior sensitivity in detecting early amyloid infiltration, particularly in AL amyloidosis, compared to LGE [5]. The ECV fraction enhances diagnostic accuracy by providing a direct measurement of matrix expansion due to amyloid deposits. In this case, the significantly elevated ECV of 52.3% offered robust evidence supporting the diagnosis of cardiac amyloidosis [6].

Furthermore, CMR identified extracardiac manifestations, including minimal pericardial effusion, bilateral pleural effusions, and perihepatic ascites, which were overlooked by echocardiography. Most notably, CMR facilitated the detection of a left atrial appendage thrombus measuring 9.6 x 8.8 mm, appearing as a signal-void area without enhancement. This underscores the capability of CMR not only to detect structural abnormalities but also to identify thrombi with high specificity, reducing the need for invasive procedures like endomyocardial biopsy in certain cases [5].

### Thrombus detection and comparison to echocardiography

The detection of a thrombus in the left atrial appendage by CMR, despite the absence of Afib, underscores the potential for rapid intracardiac thrombus formation in patients with cardiac amyloidosis. This thrombus may have developed earlier due to undetected Afib episodes before the patient's presentation. TTE is limited in its ability to detect left atrial thrombi, especially small thrombi, due to its lower sensitivity in this region. While transesophageal echocardiography (TEE) provides higher sensitivity for thrombus detection, it was not indicated in this patient, given the absence of clinical signs warranting TEE [7,8]. The noninvasive nature of CMR makes it an excellent modality for identifying both myocardial infiltration and thrombi, as seen in this case.

### Biomarker significance in cardiac amyloidosis

Elevated proBNP (13,344.2 pg/mL) and high-sensitivity troponin (57.0 pg/mL) levels were observed, both key indicators of cardiac stress. These elevations indicated cardiac injury or myocardial stress, which are frequently associated with cardiac amyloidosis. Particularly, high-sensitivity troponin serves as a poor prognostic marker in AL amyloidosis [9] and helps differentiate cardiac amyloidosis from other forms of hypertrophy [10]. ProBNP is elevated due to increased ventricular wall stress from diastolic dysfunction, which is characteristic of amyloid infiltration. Elevated troponin indicates myocardial injury or necrosis, commonly seen as amyloid deposits disrupt the myocardial architecture.

### Thromboembolic risk in cardiac amyloidosis

In addition to Afib, cardiac amyloidosis, particularly AL amyloidosis [11], independently increases thromboembolic risk through other mechanisms. One possible mechanism is atrial dysfunction or "atrial myopathy," where amyloid infiltration into the atrial myocardium impairs atrial contraction, leading to blood stasis, a key factor in thrombus formation. Furthermore, cardiac amyloidosis is often associated with a prothrombotic state, possibly due to systemic inflammation and endothelial dysfunction, further increasing the risk of thromboembolic events [12].

### CMR as a noninvasive tool for thrombus detection

CMR is a noninvasive imaging modality capable of detecting intracardiac thrombi, regardless of the underlying cause. This case also highlights the clinical importance of regular follow-up in patients with cardiac amyloidosis. Monthly visits with ECGs and biomarker testing (e.g., NT-proBNP and troponin) are recommended, along with annual echocardiograms and 24-hour Holter monitoring. The case advocates for a lower threshold for using advanced imaging techniques, such as CMR, in suspected cases of cardiac amyloidosis, as timely intervention based on accurate diagnosis can significantly alter the clinical course and improve patient outcomes.

## Conclusion

The rapid progression from MGUS to AL amyloidosis, particularly cardiac amyloidosis, is a rare but clinically significant event that challenges the traditional perception of MGUS as benign. Cardiac amyloidosis, especially in its AL form, is highly progressive and can present with intracardiac thrombi, even in the absence of overt atrial fibrillation or despite controlled rhythm. This underscores the necessity for a proactive clinical approach. CMR has demonstrated superiority over traditional echocardiography in detecting such critical conditions, making it indispensable for early detection and effective management. The findings in this case highlight the need for heightened vigilance in MGUS patients and suggest a lower threshold for employing advanced imaging in suspected cases of cardiac amyloidosis. Integrating timely CMR evaluations with early anticoagulation and rhythm control is essential to prevent severe outcomes.

This case advocates for updates in clinical practice guidelines and diagnostic protocols to include earlier use of advanced imaging, particularly CMR, in patients with MGUS at risk of progression to amyloidosis. Additionally, future studies should focus on the early use of CMR in MGUS patients and further investigate the role of advanced imaging in detecting thrombi and assessing disease progression. Implementing such strategies may lead to earlier interventions, better patient outcomes, and improved long-term management.

## Patient consent

Verbal consent was obtained from both the patient and his son. Additionally, the son completed a written consent form on behalf of the patient, providing permission for the publication of the case details, including any related medical data or images, while ensuring that patient confidentiality and anonymity are maintained.
